# Modifying the Attention Bias Test to Assess the Emotional State of Dogs

**DOI:** 10.3390/ani15060840

**Published:** 2025-03-14

**Authors:** Holly G. Molinaro, Ella Smith, Esmé Crawford-Paz Soldán, Clive D. L. Wynne

**Affiliations:** 1 Psychology Department, Arizona State University, Tempe, AZ 85281, USA; cwynne1@asu.edu; 2 Biochemistry Department, Arizona State University, Tempe, AZ 85281, USA; elsmit32@asu.edu; 3 College of Veterinary Medicine, Washington State University, Pullman, WA 99163, USA; e.crawford-pazsolda@wsu.edu

**Keywords:** canine, animal emotions, cognitive tests, human-animal interactions, animal welfare

## Abstract

The attention bias test is an established cognitive method used to evaluate emotional states in farm animals by examining their response to a stimulus under varying emotional conditions. This study adapted this test for dogs, utilizing naturally induced emotional states in place of pharmacological interventions. Dogs were emotionally primed into positive, neutral or negative emotional states through interactions with their owners before encountering a novel, mildly threatening stimulus (a fan with colorful streamers attached) in a controlled arena. Behavioral responses were observed and recorded, revealing distinct patterns linked to emotional states. Notably, dogs in the negative condition exhibited increased pacing, reduced vocalizations, and a tendency to remain near the exit door—behaviors consistent with heightened anxiety. In contrast, dogs in the positive and neutral conditions showed less pronounced behavioral differentiation. These findings highlight the potential of the attention bias test as a valuable tool for assessing emotional states in dogs. This application underscores the importance of further research to refine methodologies, deepen our understanding of canine emotions and enhance animal welfare practices.

## Simple Summary

Although people have strong intuitions about their dogs’ emotions, there is a shortage of scientific information about what dogs may be feeling. Animal emotions are difficult to study because we cannot directly ask them how they are feeling. Consequently, emotional states in animals are studied with cognitive and behavioral tests. Here for the first time, we infer emotional states in dogs using the attention bias test, which has been used in past studies with other animal species. Dogs were first emotionally primed by their owners into positive, neutral or negative emotional states and then entered a testing arena to interact with a novel stimulus. The novel stimulus was a noisy fan intended to produce mild discomfort in the dogs. We observed whether the dogs responded differently to the fan depending on the priming group they were in. We found that dogs in the negative priming group were more likely to pace, stay near the exit, and less likely to vocalize than dogs in the other groups. The behavior of dogs in the positive and neutral groups was not as distinct. These results indicate that the attention bias test is a promising tool for assessing emotional states in dogs. Development of this test can improve our understanding of dog emotions and consequently improve our ability to provide quality care to dogs.

## 1. Introduction

Understanding and accurately assessing the emotional states of animals is crucial for enhancing their care, well-being and overall welfare. However, the inability of animals to self-report their emotions presents a significant challenge in determining their emotional states in various contexts. Therefore, research into reliable methods for evaluating animal emotions is essential.

Current methods of assessing animal emotions range in both effectiveness and accuracy. Most animal emotion research focuses on human interpretations of what the animal may be feeling, which may bias conclusions. When it comes to objectively studying an animal’s emotional state, the theory of emotion one subscribes to leads to different definitions of what even constitutes an emotion or effect in animals [1]. Methods such as fMRI imaging have been used to locate brain regions associated with emotional states [2], or scientists assign behaviors to emotional states by systematically correlating observable behaviors, such as body language or vocalizations, with physiological measures (e.g., heart rate) to infer emotional states [3].

Recently cognitive methods have been developed to study an animal’s emotional states as well. These methods exploit the fact that cognitive and mental states interact [4,5]. For example, the cognitive- or judgment- bias test allows baseline emotional parameters to be measured by training animals to associate cues with positive or negative outcomes (presumably through classical conditioning of cues). For example, animals may learn that one stimulus predicts a reward while another predicts no outcome. The animal’s choices between these cues during follow-up testing indicate their current positive or negative emotional valence state. This test has been used in a variety of different species (horses [6]; rhesus macaques [7]; rats [8]; pigs [9]; ravens [10]; zoo animals including gorillas, dolphins and chimpanzees [11]; and bumblebees [12]). The cognitive approach to studying animal emotions has many benefits, as it is less invasive than neuroimaging methods and reduces the influence of human biases by relying on quantifiable behavioral indicators. If we cannot directly measure an animal’s emotional state, we can measure their cognitive processes and thus infer their emotional state because cognitive and emotional processes act on one another reciprocally.

The emotions of dogs have been studied more than those of many other species, and yet these studies are still beset by limitations, particularly the overreliance on human interpretation. Common approaches, such as analyzing facial expressions through systems like DogFACS (a standardized facial action coding system designed to objectively measure facial muscle movements in dogs, which can be linked to emotional states [13]) or measuring discrete behavioral and physiological responses to infer positive or negative emotional states, often lack definitive validation [14,15,16]. In contrast, judgment bias tasks offer a more objective method for assessing a dog’s emotional state that minimizes the influence of human biases on behavioral interpretation.

Mendl et al. (2010) used a judgment bias task where dogs were trained to discriminate between a positive (always rewarded) and a negative (never-rewarded) location [17]. During testing, their responses to ambiguous locations were measured to assess emotional states. Dogs who were reported to have high levels of separation-related behaviors performed more ‘pessimistically’ on a judgment bias task by running more slowly to an ambiguous location. In these tasks, ‘pessimism’ refers to a tendency to interpret ambiguous cues as predicting negative outcomes, which may indicate a negative emotional state. In contrast, another study Müller et al. (2012) [18] found that a brief owner absence did not prompt a pessimistic bias in dogs. Using a similar methodology to [17], Müller et al. (2012) [18] used a within-subject design, testing the same dogs in both owner-present and owner-separated conditions. These contrasting results showcase how differing contexts and conditions within a cognitive bias test can produce diverse results. Müller et al. (2012) [18] also noted that there were extreme differences between individual dogs.

As detailed in several of the studies above, the context of the judgment bias task can influence the results. Many of these experiments rely on the dog’s ability to memorize the locations of the positive and negative stimuli. Therefore, judgment bias tests also rely on the dogs’ spatial learning ability [19,20].

The judgment bias task separates emotional valence (positive or negative feelings) from arousal (intensity of emotion), allowing researchers to differentiate between emotional states such as excitement and anxiety, which is crucial for accurate emotion assessment [21]. According to the American Psychological Association Dictionary of Psychology, ‘anxiety’ is an emotional state. However, there are also limitations in the experimental design, setup and analysis. For example, Starling et al. (2014) [22] used a portable automated device to test judgment bias in dogs. While finding strong individual differences between the dogs, there was also a tendency for the dogs to be less likely to approach any of the stimuli as testing proceeded. In addition, these judgment bias tests require lengthy training periods for the animals and the necessary presence of a human researcher during testing, which biases the results for species such as dogs who find humans an inherently pleasurable stimulus, refs. [23,24,25] is a further problem. These limitations prompted us to seek a novel task for the assessment of emotions in dogs.

The attention bias test is a promising new task in the animal emotion and cognition field [4]. The attention bias test is considered a cognitive task because it assesses how animals allocate their attention to different types of stimuli, revealing underlying emotional states and cognitive processes. Attention is a fundamental cognitive function, shaping how animals perceive and interact with their environment [4]. This test assesses an animal’s focus on a particular stimulus, typically under the influence of an induced emotional state. The underlying principle involves placing the animal in either a positive or negative emotional state, followed by the introduction and subsequent removal of a perceived threat. The animal’s behavior after the threat is withdrawn provides insight into its emotional condition, revealing species-specific emotional indicators. For example, an animal in a negative emotional state is expected to remain anxious or vigilant toward the location of the threat even after it has been removed. In contrast, an animal in a positive emotional state is likely to return to baseline behaviors, such as exploration or normal activities, once the threat is no longer present.

To date, the attention bias test has been trialed in livestock including pigs [26], sheep [27,28], and cows [29] as well as with lab-housed macaques [30]. Lee et al. (2016) [27] demonstrated that the attention bias test can differentiate high and low anxiety states in sheep. Sheep were either given 1-methyl-chlorophenylpiperazine to increase or diazepam to decrease anxiety. Responses of the sheep were then measured in reaction to the presence of a threat, in this case, a dog. It was found that sheep in the high anxiety state paid the most attention to the area where the dog had been once it was removed. Lee et al. (2018) [29] used similar attention bias test methods with pharmacologically induced states with cattle and found that those cows in the high anxiety condition also displayed increased vigilance towards a threat. However, the authors noted that the test may not be useful for positive emotional states, but only because most research so far has only looked at its effectiveness in negative ones. This method has also been used with lab-housed macaques to measure welfare after a surgical procedure [31]. The study demonstrated that the test could detect emotional differences among the monkeys. However, the responses varied based on the duration of threat exposure, with shorter threat durations eliciting greater attention.

Past studies have demonstrated how the attention bias test can be used to measure negative affective states, with the potential to detect positive ones. However, in all prior studies, affective states were artificially induced with either drugs or surgery. Neither approach is appropriate for pet dogs, thus, the innovation of the current study lies in assessing a novel, non-captive, animal species and testing whether the task is effective when the emotional state is induced naturally rather than with surgery or drugs. Unlike cognitive bias tasks, which can be influenced by factors such as memory and human presence, the attention bias test allows for a clearer separation of emotional valence and arousal and minimizes human-induced biases. The aim of this study was to modify the attention bias test for dogs, by placing dogs in naturally induced positive, neutral and negative emotional states. We would then expose them to a stressor and observe their behavioral responses after the threat has been removed, allowing us to assess how their prior emotional state manifests. Applying this method to dogs could significantly enhance our ability to evaluate their welfare and emotional well-being in a more accurate and scientifically sound manner. In addition, if we are able to establish ways in which the test can and must be modified for diverse species, we can demonstrate how others can adapt this test for other animals, thus ultimately helping a variety of animals under human care.

## 2. Materials and Methods

This study was approved by the Institutional Animal Care and Use Committee (IACUC) of Arizona State University (protocol number: 23-1980R).

### 2.1. Subjects

45 pet dogs of various sizes, breeds and ages were tested (see Supplementary Materials for complete demographic info on dogs). All dogs were recruited by word of mouth in the Tempe, Arizona area and all owners volunteered their dogs to participate.

### 2.2. Materials and Setup

The experimental setup consisted of two distinct areas: one designated for the emotional induction phase and another for the attention bias testing.

**Figure 1:**
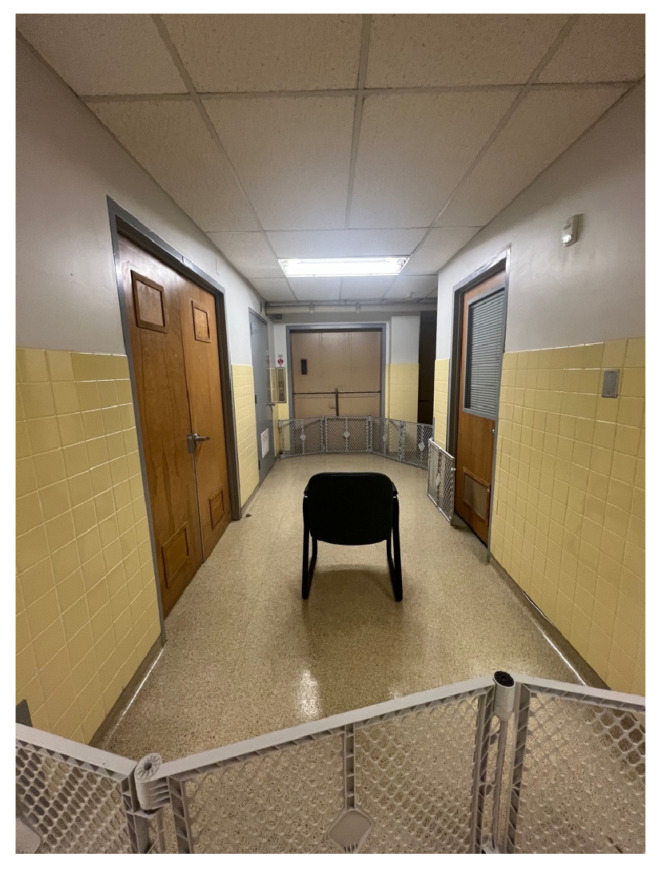
Emotional induction phase arena.

The emotional induction area was a sectioned-off portion of a hallway located 1.5 m outside the attention bias testing arena (see Figure 1). This space, measuring approximately 3 by 4.5 m, was enclosed using dog gates on either end to ensure the dog and owner remained contained. The setup provided a quiet, distraction-free environment, shielding the dog from external sounds or foot traffic within the building. At the center of this area, a chair was placed for the owner to sit on during the conditioning period.

**Figure 2:**
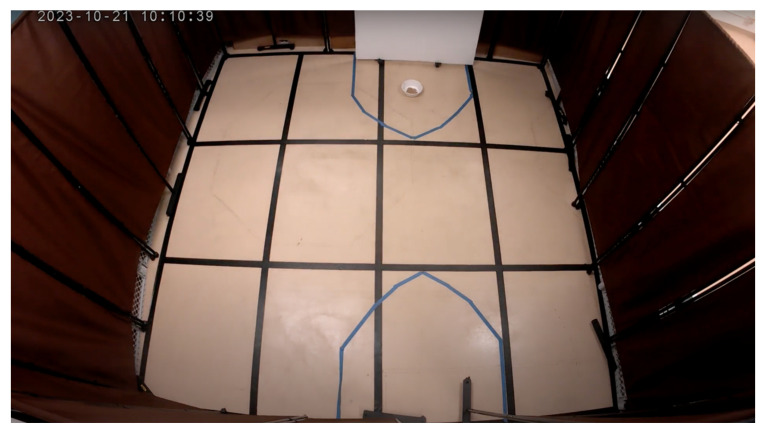
Overhead camera view of attention bias arena.

The attention bias testing area was in an adjacent room connected to the hallway. Upon entering the testing space, one encountered four 1.8-m-tall brown room dividers partitioning off a space 3-m by 4-m square (Figure 2). Two openings were left in the enclosure: one for entry and another, directly opposite, to introduce the threatening stimulus. To secure the entry point, a white 1-m by 1.5-m Styrofoam board was positioned in front of the door, preventing dogs from exiting. The opposite opening, used to present the threatening stimulus, featured a small pulley system attached to the ceiling. A rope connected to an identical white 1-m by 1.5-m Styrofoam rectangle allowed researchers outside the testing arena to manipulate the opening remotely. The arena floor was marked with a 1-m by 1-m grid using black duct tape, and blue duct tape was used to create 1-m diameter semicircles near the door and around the threatening stimulus area.

**Figure 3:**
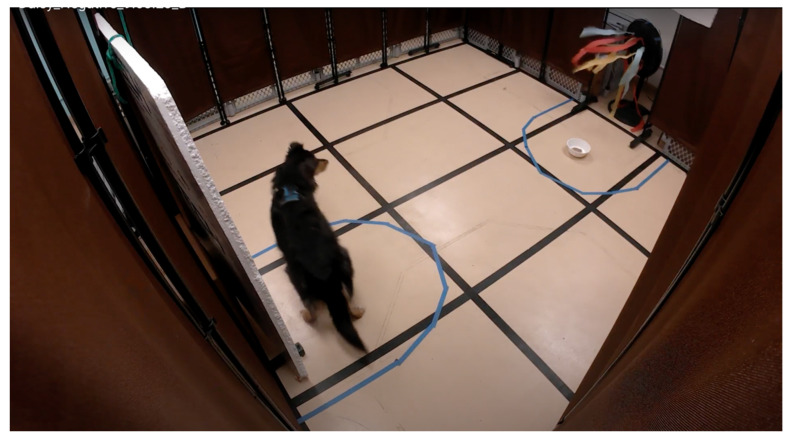
Side camera view of attention bias arena with dog and fan on.

The threatening stimulus was a black standing fan, modified with colorful streamers and cardboard attachments to produce loud noises and visually disorienting effects (Figure 3). In the semicircle surrounding the fan, a disposable cardboard food bowl was taped to the floor, containing approximately half a can of either chicken or beef Blue Buffalo wet dog food.

Two cameras were installed to monitor the dogs during testing. One was mounted above the entrance door which recorded the dog for behavioral coding. The other was placed in the right-hand corner of the arena for owners to watch their dogs. Both cameras provided live-stream footage, enabling both researchers and owners to observe the dogs throughout the procedure.

### 2.3. Procedure

Before the experiment, each dog was randomly assigned to one of three emotional state conditions: positive, negative, or neutral. A prior power analysis assuming a medium effect size with 80% power at a significance criterion of α = 0.05, indicated 15 dogs per group would be needed to detect statistically significant differences. Upon arrival at the lab, the dog’s owner completed a consent form, and the experimental procedure was explained in detail, including the roles of the owner and the researchers. Owners were informed of their dog’s assigned emotional condition before arrival, allowing them to bring specific stimuli to facilitate emotional induction (e.g., nail clippers for the negative condition or favorite toys for the positive condition). After introductions, the dogs were offered water ad libitum, but no water was provided during the five-minute testing session.

For dogs in the positive group, owners engaged in activities they believed would evoke a positive state in their dog, such as petting, praising, offering treats, or playing. Dogs in the neutral group served as the control, with owners instructed to maintain a calm and neutral demeanor, involving occasional petting and minimal interaction. For the negative group, owners employed actions they believed would be likely to induce a negative emotional state in their dog, such as verbally scolding, ignoring, or presenting disliked items.

The experiment began with the owner and dog entering the emotional induction area, while Experimenter 1 (E1) sat just outside the dog’s field of view. E1 initiated a two-minute timer, during which the owner performed the emotional induction activities. After two minutes, E1 instructed the owner to stop and escort the dog to the attention bias testing arena. At the entrance, the owner unclipped the dog’s leash, allowing the dog to enter the arena independently and alone.

Once the dog entered, E1 positioned the Styrofoam rectangle in front of the opening, and the lab door was closed. Experimenter 2 (E2) started the three-minute testing timer from an adjacent room outside the attention bias arena. After 15 s, Experimenter 3 (E3) activated the fan—the threatening stimulus—for 10 s, following the removal of the Styrofoam cover by E2. The fan was then turned off, and the Styrofoam cover was replaced using the pulley system. The dog remained in the testing arena for the full three minutes, after which E1 reopened the door, and the dog was reunited with its owner.

Following the testing session, dogs were again offered water, received a certificate, and allowed to leave the lab. Between each individual testing session, the testing arena was cleaned and disinfected to limit the carry-over of odors from previous sessions and fresh food bowls were provided to ensure a consistent and sanitary environment for all participants. Throughout the experiment, owners could monitor their dogs via a live video feed, and any visible signs of distress or owner concern would have resulted in the immediate cessation of the test (this never occurred). All sessions were recorded for subsequent analysis.

### 2.4. Data Analysis

Data were analyzed using Version 29.0.2.0 SPSS. *p* values less than 0.05 were considered significant. All data were checked for normality through visual assessment and the Shapiro–Wilks test. Count data were analyzed using a generalized linear model with a negative binomial distribution to account for over-dispersion (all variables are in Table 1). Demographic variables and their effect on the other variables were analyzed with one-way ANOVAs. Behavior data were analyzed with either one-way ANOVAs or Kruskal–Wallis non-parametric ANOVAs, depending on whether the data were normally distributed or not (all variables are in Table 2). Non-parametric behavioral data included: seconds attempting to leave, seconds within the fan 1 m semicircle while the fan was on and while the fan was off, seconds within the door 1 m semicircle while the fan was on, seconds laying down while the fan was off, total time spent eating, total time sitting or lying down, and total time spent looking at the fan or board while the fan was on or after it was covered up. All other behavioral data were normally distributed. Latency data were analyzed with a Cox regression analysis (see Supplementary Materials). Due to the high levels of variance among the groups in the behavioral data, Levene’s test was also used to see whether the variance differed by group. Finally, Fisher’s exact tests and binomial tests were used to analyze differences across groups.

A composite score was created for measures of resting behaviors and for anxiety behaviors. The anxiety score for the dogs was created by combining (summing) the following variables: seconds attempting to leave, total seconds in a 1-m-diameter semicircle around the exit door, and total time spent looking at the door. The resting score summed the following variables: total seconds in a 1-m-diameter semicircle around the fan 1 m, total time spent looking at the fan and board, and total seconds sitting and lying down. These variables for the compositive score were chosen because they did not overlap with other variables, and they related to either the dog being in a relaxed or anxious state. They were both analyzed with one-way ANOVAs.

## 3. Results

### 3.1. Test Effectiveness

First, binomial tests were carried out to investigate whether the attention bias test with the threatening fan was successful. If the phase of the test where the fan was uncovered and on provided a useful measure of stress response, we would expect that significantly more dogs would be in the 1-m^2^ semicircle around the door while the fan was on than would be expected by chance. Since the semicircle around the door occupied 1/12th of the total 12-m^2^ floor area, we set the chance probability at 0.083. A total of 13 of the 15 (87%) dogs in the negative group were within the door semicircle when the fan was on (binomial *p* < 0.001). A total of 9 of the 15 (60%) dogs in the positive group were within the door semicircle when the fan was on (binomial *p* < 0.001). Only 6 out of the 15 dogs (40%) in the neutral group were in the door semicircle when the fan was off, which was significantly fewer than those outside of it (binomial *p* < 0.001).

This indicates the fan as a threat provided a stress response for those in the negative and positive group only.

If the phase after the fan stopped was a measure of stress resilience (recovery after a disturbance), then we would expect to see more dogs approach the food bowl after the fan was turned off and covered than would be expected by chance in this phase. Again, since the semicircle with the food bowl and close to the fan occupied 1/12th of the total 12-m^2^ floor area, we set the chance probability at 0.083.

A total of 11 of the 15 (73%) dogs in the negative approached the food bowl after the fan was turned off (binomial *p* < 0.001). A total of 11 of the 15 (73%) dogs in the positive group approached the food bowl after the fan was turned off (binomial *p* < 0.001). A total of 9 out of the 15 dogs (60%) in the neutral group approached the food bowl after the fan was turned off (binomial *p* < 0.001). Overall, 31 of the 45 dogs (69%) approached the food bowl after the fan had been turned off (binomial *p* < 0.001).

In addition, if the phase after the fan turned off was a measure of stress resilience, then overall we would expect to see more dogs in the fan 1-m semicircle area once the fan was off and covered than would be expected by chance in this phase.

A total of 12 of the 15 (80%) dogs in the negative were in the fan semicircle after the fan was turned off (binomial *p* < 0.001). A total of 12 of the 15 (80%) dogs in the neutral were in the fan semicircle after the fan was turned off (binomial *p* < 0.001). Finally, 10 of the 15 (67%) dogs in the negative were in the fan semicircle after the fan was turned off (binomial *p* < 0.001). Overall, 34 dogs (76%) were in the fan semicircle after the fan had been turned off, while 11 did not enter (binomial *p* < 0.001).

### 3.2. Count Data

**Figure 4:**
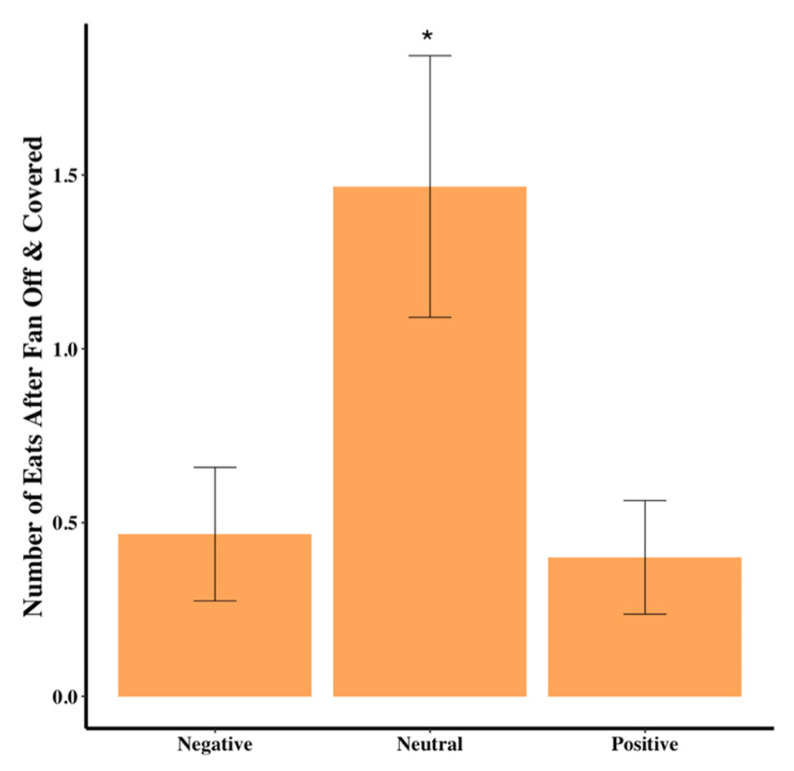
Average number of times the dog ate after the fan was off and covered. Asterisk (*) denotes significant differences from the other two conditions. Error bars show standard errors.

The number of barks was significantly different among the groups: dogs in the negative group were about half as likely to bark than those in the neutral group (χ^2^(2) = 6.07, *p* = 0.048, Table 1). The number of whines was significantly different among the groups, with dogs in the negative group about half as likely to whine as in the positive group (χ^2^(2) = 9.40, *p* = 0.009, Table 1). The number of times the dog ate some food out of the bowl after the fan was turned off and covered was significantly different among the groups, with the neutral group almost four times as likely to eat compared to the positive and negative groups (χ^2^(2) = 6.721, *p* = 0.035, Table 1, Figure 4).

### 3.3. Behavioral Data

Total time spent walking in the testing area was marginally significantly different among groups (F_2,44_ = 2.95, *p* = 0.06, Table 2); a post hoc test revealed dogs in the negative group spent more time walking than in the positive group. When analyzing the variable of seconds attempting to leave the testing area, dogs in the negative group had more variation than those in the positive group (F_2,42_ = 4.19, *p* = 0.022). When analyzing the variable of total time spent near the fan and door, dogs in the neutral group had more variation than dogs in the negative group (F_2,42_ = 3.47, *p* = 0.041).

**Figure 5:**
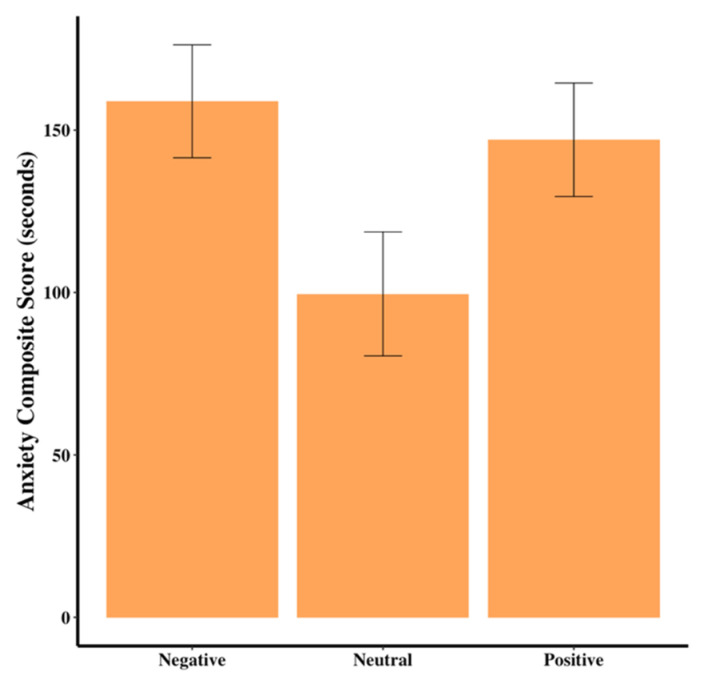
Anxiety composite score for the dogs across the three groups. Error bars show standard errors.

Dogs in the neutral group had marginally lower anxiety scores than dogs in the negative group (F_2,44_ = 3.03, *p* = 0.06, Table 2, Figure 5). There were no differences among the groups for resting scores (*p* > 0.05).

### 3.4. Latency Data

There were no significant differences among the latency data (all *p* > 0.05, see Supplementary Materials).

### 3.5. Demographic Variables

Age, sex and breed of the dog (purebred or mixed) did not significantly impact any of the variables overall (all data in Supplementary Materials).

### 3.6. Other Trends

A 2 × 3 Fisher’s exact test was used to determine whether there was a significant association between groups and whether all zones were entered. There was a marginally statistically significant association between the two variables (*p* = 0.06). Therefore, follow-up 2 × 1 Fisher’s exact tests were used to analyze between-group differences. There was no significant difference between the neutral and positive groups in terms of zones entered (*p* = 1.0) nor between the negative and neutral groups (*p* = 0.11). However, there was a statistically significant difference between the positive and negative groups, in that more dogs in the negative group entered all zones than in the positive group (*p* = 0.05).

A 2 × 3 Fisher’s exact test was also used to determine whether there was a significant association between groups and whether the dogs were in the door 1 m semicircle while the fan was on. There was a statistically significant association between the two variables (*p* = 0.04). Therefore, follow-up 2 × 1 Fisher’s exact tests were used to analyze between-group differences. There was no significant difference between the neutral and positive groups in whether the dog was in the door 1 m semicircle (*p* = 0.47) nor the negative and positive groups (*p* = 0.22). However, there was a statistically significant difference between the neutral and negative groups, in that more dogs in the negative group were in the door semicircle than in the neutral group (*p* = 0.02).

**Figure 6:**
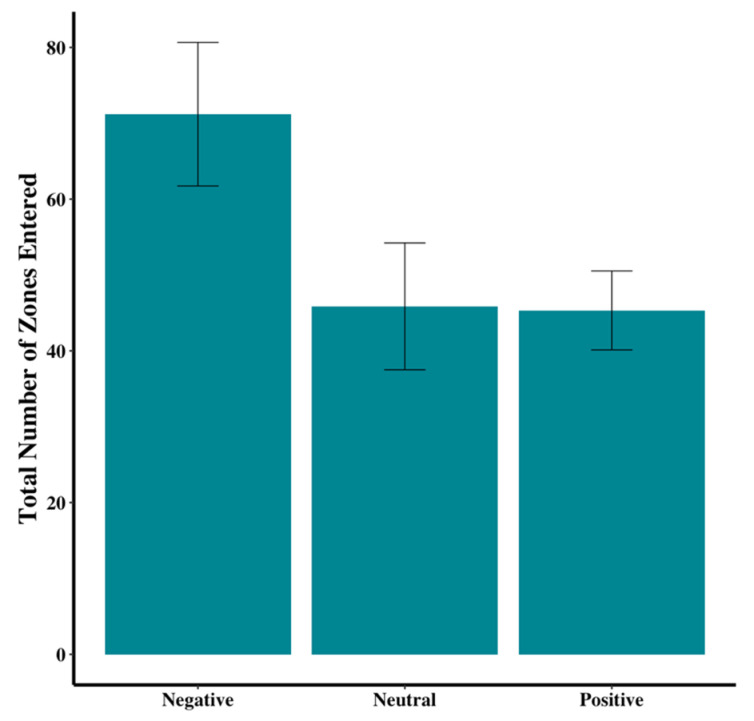
Total number of zones the dog entered. Error bars show standard errors.

**Figure 7:**
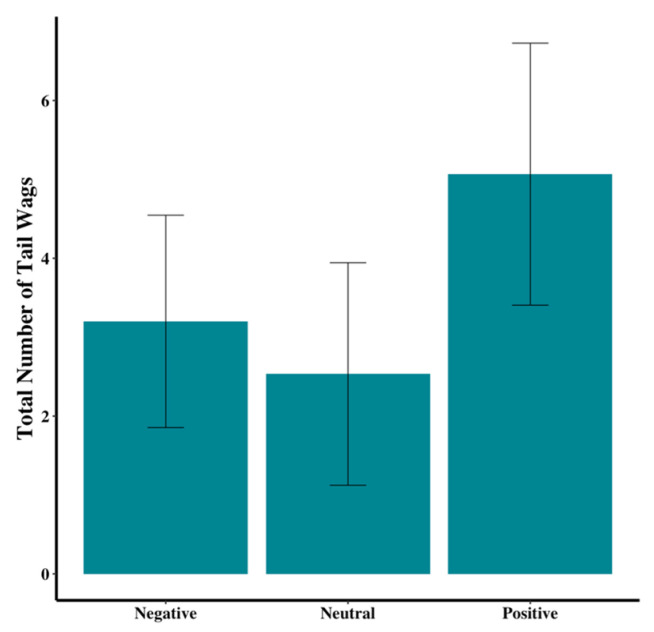
Total number of times dog wagged tail. Error bars show standard errors.

**Figure 8:**
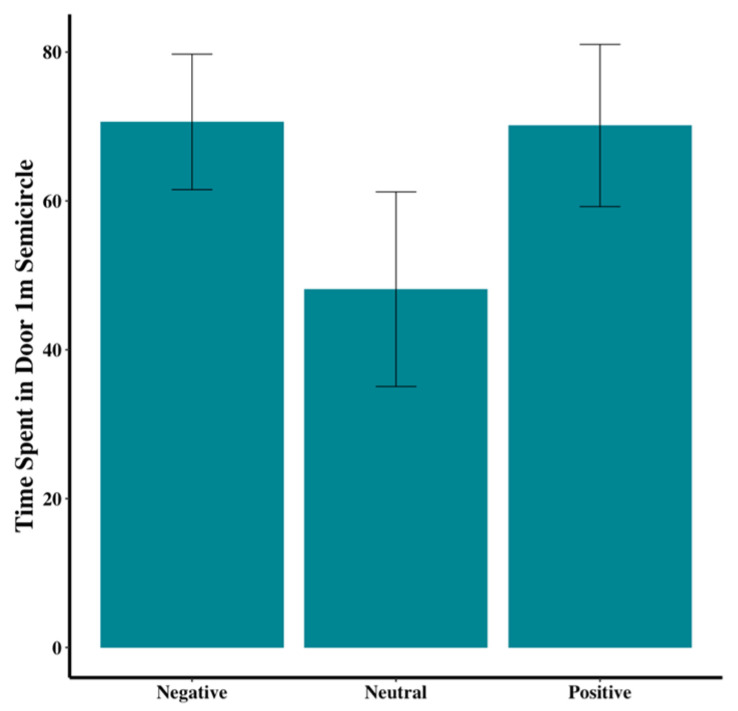
Total time (seconds) dog spent by door to exit. Error bars show standard errors.

While not significant, visual inspection suggested trends in the data that could inform future research. Dogs in the negative group tended to enter more floor zones than those in the neutral and positive groups (χ^2^(2) = 2.01, *p* = 0.37; Figure 6). In addition, tail wagging appeared to be more prevalent in the positive group, compared to the other two groups (χ^2^(2) = 2.93, *p* = 0.23; Figure 7). Dogs in the neutral group tended to spend less time within the 1-m-radius semicircle at the door than those in the positive or negative groups (F_2,44_ = 1.67, *p* = 0.20; Figure 8).

**Figure 9:**
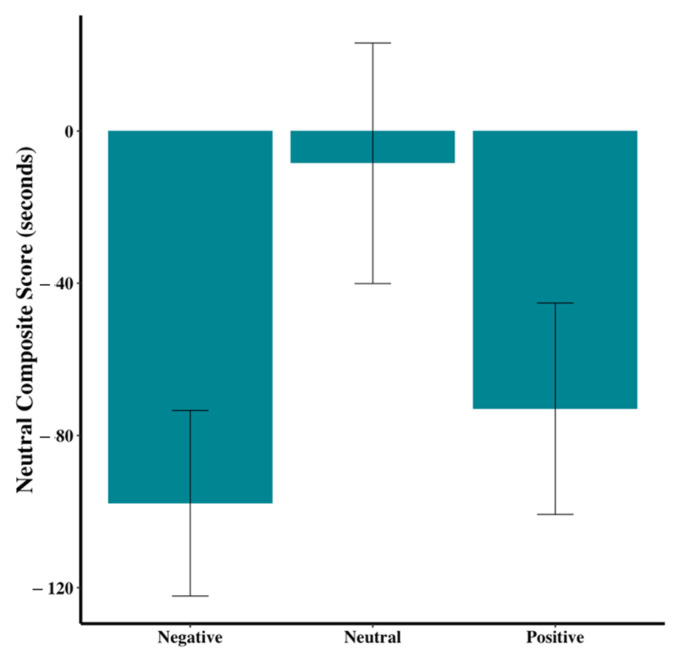
Resting minus anxiety composite score across the three groups. Error bars show standard errors.

An overall neutrality score for each dog was created by subtracting the anxiety score from the resting score and then comparing across the three groups. While the differences were not significant, Figure 9 (F_2,44_ = 2.70, *p* = 0.08) shows a trend for dogs in the neutral group to have different neutrality scores than those in the positive or negative groups.

## 4. Discussion

The current study aimed to evaluate the feasibility of an attention bias test to measure emotional states in pet dogs under naturally induced conditions. Unlike previous studies on livestock or lab-housed animals that relied on pharmacological manipulations to induce emotional states, this experiment sought to induce positive, neutral and negative emotional contexts through interactions between dogs and their owners. The results revealed several promising trends, albeit with limitations, suggesting that the attention bias test could offer a valid and objective tool for assessing canine emotions.

We first investigated whether the attention bias test with the threatening fan was successful. Results showed that this test worked as both a test of stress and stress resilience. The behavior of dogs during the fan stimulus phase also supports the efficacy of the attention bias test in eliciting a stress response. Specifically, the majority of dogs in both the negative and positive groups positioned themselves within the door semicircle during the fan phase, indicating heightened attention toward the perceived threat. This pattern was not evident in the neutral group. This may be because the neutral group experienced less emotional stimulation compared to the positive and negative groups during the emotional induction phase. Post-stimulus behaviors, such as an approach to the food bowl and time spent in the fan semicircle, provide a measure of stress resilience. A substantial proportion of dogs approached the food bowl and entered the fan semicircle after the fan was turned off, reflecting a return to baseline exploratory behavior. These findings suggest the test is effective in capturing the recovery phase of emotional regulation. Resilience can be a sign of positive welfare, and especially in dogs showcases positive emotional states [32,33]. In terms of dog welfare, tests designed to assess fear and anxiety can also serve as valuable tools for evaluating stress resilience, ultimately helping to identify strategies that promote positive emotional states in dogs [34].

One major benefit of this test is the objective measures of the behaviors associated with positive, neutral and negative states. Presently, although people have powerful intuitions about dogs’ expression of emotions, objective measures are absent from the scientific literature. This test can be used to uncover specific emotional behaviors without human bias. A similar advantage of the attention bias test was observed in cattle. Lee et al. (2018) [29] examined various behaviors, including flight speed and crush score—two metrics widely used in the industry to assess emotional states in cattle. Interestingly, the study revealed that these measures were not linked to negative emotional states, challenging previous assumptions.

Overall, we found there were some differences among the dogs in the negative group compared to the other two groups. Dogs in the negative condition exhibited a few distinct behaviors, including increased pacing as shown by a tendency to enter all zones marked in the testing arena, reduced vocalizations (fewer barks and whines), and greater proximity to the exit door during the fan stimulus. What makes this test particularly valuable is its ability to identify and measure behaviors associated with emotional states rather than relying on preconceived assumptions about what those behaviors should be. Traditional methods often start by assigning certain actions or physiological responses to specific emotions, potentially introducing bias. In contrast, the attention bias test allows the behaviors themselves to reveal the animal’s emotional state through observation and analysis. Therefore, we propose that pacing, fewer vocalizations and seeking proximity to an exit are behavioral indicators of negative affect in dog.

These findings are consistent with previous research demonstrating that the attention bias test indicates how negative emotional states enhance vigilance and stress behaviors [26,30,35]. Lee et al. (2016) [27] employed the attention bias test with sheep given two different drugs to induce heightened or depressed anxiety states. Although they did not incorporate positive emotional states for comparison, they demonstrated the test’s effectiveness in identifying negative emotional behaviors in the sheep, such as increased vigilance.

In our study, dogs in the positive and neutral groups displayed less distinct behavioral differentiation, indicating either that the emotional induction protocols may require refinement to achieve clearer group distinctions, or the attention bias test is not sensitive enough for positive emotional conditions. Past research points to the latter. Monk et al. (2020) [28] used the attention bias test with sheep in four different pharmacologically induced conditions: happy, calm, anxious and a control. It was found that there were behavioral differences between the anxious and control groups, yet no differences among the positive groups. It was hypothesized that since the premise of the attention bias test involves novelty and isolation, it may only be able to capture negative emotional states.

There were several limitations to this current study. An important one was the reliance on naturally induced emotional states, which differs from previous studies where affective states were artificially induced via drugs or surgery [27,31]. While our approach makes the findings more applicable to real-world settings, it also introduces variability due to the difficulty in consistently manipulating emotional states. Nonetheless, the natural induction of emotional states offers a more humane and practical alternative for pet dogs, making this approach particularly valuable for pet animal welfare research. Future studies could introduce consistency by using stricter instructions for owners to follow for each of the emotional conditions. In addition, the interactions between owner and dog during this induction phase could also be filmed and analyzed, which would yield more consistent information on what exactly induces positive or negative emotional states in the dogs. In addition, expanding the sample size could improve the generalizability of findings and help control for individual differences.

Furthermore, dogs could have been first assessed with validated behavioral scales, such as the CBARQ or even a veterinary specialist, as dogs with behavioral abnormalities may have affected the results. Finally, modifying the test to include more engaging and enriching stimuli, or exploring additional behaviors (e.g., play behaviors), could enhance its sensitivity to positive effects. Although some results did not reach statistical significance, trends in the data, such as increased tail wagging in the positive group and more zone exploration in the negative group, suggest the value of further exploration. These trends could inform future studies, particularly regarding how different emotional states manifest in distinct behavioral patterns. For instance, the tail-wagging trend could indicate heightened positive affect, while increased pacing might reflect anxious vigilance and overall negative affect.

## 5. Conclusions

In conclusion, this study highlights the potential of the attention bias test as a quick and straightforward method for assessing positive and negative emotional states in dogs, as it offers several advantages over traditional emotion assessment methods. As this is the first time the test has been adapted for canine subjects, further research is needed to refine its methodology, address individual variability and explore natural ways to induce emotional states. These advancements would enhance the test’s reliability and effectiveness, broadening its application to various emotional contexts in addition to its practical application for both dog owners and dog professionals. By developing more precise approaches, we can deepen our understanding of animal emotions and contribute to improved welfare practices for dogs and other species under human care.

## Figures and Tables

**Table 1:** Mean ± SEM of all count data for dogs in each conditioned group during the attention bias test. Data represents the entire test period.

Count Data	Negative	Neutral	Positive	Test Value	*p* Value
# of Food Approaches	2.53 ± 0.74	1.87 ± 0.64	1.20 ± 0.42	χ^2^(2) = 2.60	0.27
# of Vocalizations	8.00 ± 3.08	9.6 ± 6.84	12.27 ± 4.17	χ^2^(2) = 1.25	0.53
# of Eats Before Fan ON	0.73 ± 0.18	0.80 ± 0.17	0.73 ± 0.23	χ^2^(2) = 0.03	0.98
# of Eats After Fan OFF	0.47 ± 0.19 ^A^	1.47 ± 0.43 ^B^	0.40 ± 0.16 ^A^	χ^2^(2) = 6.72	0.035 *
# of Total Zones Entered	71.20 ± 9.46	45.89 ± 8.40	45.33 ± 5.20	χ^2^(2) = 2.01	0.37
# of Tail Wags	3.20 ± 1.35	2.53 ± 1.41	5.07 ± 1.66	χ^2^(2) = 2.93	0.23
# of Barks	3.33 ± 1.62 ^A^	8.13 ± 6.77 ^B^	7.73 ± 3.63 ^B^	χ^2^(2) = 6.07	0.048 *
# of Whines	2.40 ± 1.59 ^A^	1.20 ± 1.13 ^A^	4.73 ± 1.99 ^B^	χ^2^(2) = 9.40	0.009 *
# of Eats While Fan ON	0.13 ± 0.09	0.53 ± 0.19	0.43 ± 0.22	χ^2^(2) = 92.58	0.28

Note. Different letters denote significant differences. Asterisks (*) indicate a significant result.

**Table 2:** Mean ± SEM of all behavioral data for dogs in each conditioned group during the attention bias test. Data represents the entire test period.

Behavioral Data (Seconds)	Negative	Neutral	Positive	Test Value	*p* Value
Attempting to leave	16.30 ± 4.66	7.68 ± 3.06	11.02 ± 2.55	χ^2^(2) = 3.86	0.15
Looking at fan while ON	7.51 ± 0.99	9.40 ± 1.28	8.75 ± 1.16	F_2,44_ = 1.16	0.33
Looking in fan direction once OFF	17.60 ± 2.35	21.56 ± 4.95	18.86 ± 3.34	F_2,44_ = 0.30	0.74
Within fan 1 m while ON	3.48 ± 1.45	5.81 ± 1.62	3.32 ± 1.46	χ^2^(2) = 1.42	0.49
Within fan 1 m once OFF	16.43 ± 4.87	32.61 ± 11.22	18.33 ± 9.20	χ^2^(2) = 1.77	0.41
Within door 1 m while ON	4.64 ± 1.13	3.63 ± 1.48	4.37 ± 1.38	χ^2^(2) = 2.34	0.30
Within door 1 m once OFF	70.62 ± 8.66	44.50 ± 11.94	65.76 ± 10.09	F_2,44_ = 1.81	0.18
Laying down once OFF	9.49 ± 8.96	4.53 ± 3.75	4.26 ± 3.67	χ^2^(2) = 0.18	0.92
Total time walking	57.68 ± 7.81 ^A^	38.74 ± 8.21 ^AB^	34.42 ± 5.24 ^B^	F_2,44_ = 2.95	0.06
Total time eating	15.22 ± 5.75	34.17 ± 12.10	20.24 ± 9.35	χ^2^(2) = 1.78	0.41
Total time looking at door	67.30 ± 10.07	43.76 ± 9.10	65.87 ± 10.18	F_2,44_ = 1.82	0.18
Total time sitting or lying down	14.87 ± 9.51	21.78 ± 11.71	21.78 ± 11.71	χ^2^(2) = 0.66	0.72
Total time looking at fan/board while ON and afterwards OFF	26.27 ± 2.84	30.91 ± 5.85	30.63 ± 5.26	χ^2^(2) = 0.8	0.96
Resting Composite Score	61.05 ± 11.20	91.10 ± 16.85	74.05 ± 13.55	F_2,44_ = 2.70	0.08
Anxiety Composite Score	158.86 ± 17.39 ^A^	99.58 ± 10.11 ^B^	147.03 ± 17.48 ^AB^	F_2,44_ = 3.03	0.06

Note. Different letters denote significant differences.

## Data Availability

The raw data supporting the conclusions of this article can be found in the Supplementary Materials.

## Supplementary Materials

The following supporting information can be downloaded at: https://www.mdpi.com/article/10.3390/ani15060840/s1, Table S1: 45 pet dogs demographics (* Now attached at the end of this template). Table S2: Latency Data. Table S3: Demographic Variable Data.
