# Supplementary material for: Modifying the Attention Bias Test to Assess the Emotional State of Dogs
Source: Animals (Basel). 2025 Mar 14;15(6):840. doi: 10.3390/ani15060840 (PMC11939796; doi:10.3390/ani15060840)
Supplement: Supplementary file 1 [file animals-15-00840-s001.zip › animals-3478674-supplementary.pdf]

Modifying the Attention Bias Test to Assess the Emotional State of Dogs
Molinaro et al.
Supplementary Materials

Table S1. 45 Pet Dogs Demographics.

Dog Name	Age	Sex	Test Condition
Aaloo	2-5 years old	M	Neutral
Anthony	2-5 years old	M	Neutral
Athena	2-5 years old	F	Negative
Azul	5-8 years old	F	Negative
Bailey	5-8 years old	F	Positive
Bean	2-5 years old	F	Positive
Bella	9-12 years old	F	Negative
Bisky	2-5 years old	M	Negative
Chill	2-5 years old	M	Negative
Chrysanthemum	5-8 years old	F	Neutral
Daisy	2-5 years old	F	Negative
Dala	5-8 years old	F	Positive
Dana	5-8 years old	F	Positive
DC	5-8 years old	M	Negative
Derrick	5-8 years old	M	Negative
Doja	0-1 years old	F	Neutral
Eevee	2-5 years old	F	Positive
Ellie	2-5 years old	F	Neutral
Francis	2-5 years old	M	Neutral
Frisky	9-12 years old	M	Neutral
Halite	2-5 years old	F	Positive
Honey	2-5 years old	F	Neutral
Huckleberry	2-5 years old	M	Positive
Hunter	5-8 years old	M	Neutral
Juice	9-12 years old	F	Negative
Maggie	12 years old +	F	Negative
Marti	0-1 years old	M	Neutral
Marty	2-5 years old	M	Positive
Nico	2-5 years old	M	Neutral
Nikki	12 years old +	F	Neutral
Nitro	2-5 years old	F	Neutral
Norie	9-12 years old	F	Positive

P	2-5 years old	F	Neutral
Pitufa	2-5 years old	F	Negative
Reese	2-5 years old	F	Negative
River	12 years old +	F	Positive
Roscoe	5-8 years old	M	Negative
Rose	2-5 years old	F	Negative
Rusty	12 years old +	M	Negative
Sabina	5-8 years old	F	Neutral
Sperry	9-12 years old	F	Positive
Wiley	0-1 years old	M	Positive
Winnie	2-5 years old	F	Positive
Winnie	5-8 years old	F	Positive
Wyatt	2-5 years old	M	Positive

Table S2. Latency Data.

Latency Data	Test Value	P Value
Latency to First Bite	$\chi^2(1) = 0.007$	0.93
Latency to enter fan 1m	$\chi^2(1) = 1.473$	0.23
Latency to finish eating	$\chi^2(1) = 0.001$	0.97
Latency to first approach of food bowl	$\chi^2(1) = 0.986$	0.32
Latency to first approach food bowl after fan has been turned OFF and is covered	$\chi^2(1) = 0.446$	0.50

Table S3. Demographic Variable Data.

Variables	Age	Sex	Breed
# of Food Approaches	$F(4,42) = 1.690$, $p = 0.17$	$F(1,44) = 1.260$, $p = 0.27$	$F(1,44) = 0.048$, $p = 0.83$
# of Vocalizations	$F(4,42) = 0.069$, $p = 0.99$	$F(1,44) = 1.156$, $p = 0.29$	$F(1,44) = 0.695$, $p = 0.41$
# of Eats Before Fan ON	$F(4,42) = 0.459$, $p = 0.77$	$F(1,44) = 0.120$, $p = 0.73$	$F(1,44) = 0.004$, $p = 0.95$
# of Eats After Fan OFF	$F(4,42) = 0.883$, $p = 0.48$	$F(1,44) = 0.591$, $p = 0.45$	$F(1,44) = 1.798$, $p = 0.19$
# of Total Zones Entered	$F(4,42) = 0.207$, $p = 0.93$	$F(1,44) = 0.612$, $p = 0.44$	$F(1,44) = 2.044$, $p = 0.16$
# of Tail Wags	$F(4,42) = 1.92$, $p = 0.13$	$F(1,44) = 0.009$, $p = 0.92$	$F(1,44) = 0.859$, $p = 0.36$
# of Barks	$F(4,42) = 0.344$, $p = 0.85$	$F(1,44) = 1.188$, $p = 0.28$	$F(1,44) = 0.446$, $p = 0.51$
# of Whines	$F(4,42) = 0.957$, $p = 0.44$	$F(1,44) = 0.001$ $p = 0.97$	$F(1,44) = 0.355$, $p = 0.56$
# of Eats While Fan ON	$F(4,42) = 1.34$, $p = 0.27$	$F(1,44) = 0.972$, $p = 0.33$	$F(1,44) = 0.670$, $p = 0.42$
Attempting to leave	$F(4,42) = 0.378$, $p = 0.82$	$F(1,44) = 0.035$, $p = 0.85$	$F(1,44) = 0.779$, $p = 0.38$
Looking at fan while ON	$F(4,42) = 0.528$, $p = 0.72$	$F(1,44) = 0.082$, $p = 0.78$	$F(1,44) = 0.699$, $p = 0.41$

Looking in fan direction once OFF	F(4,42) = 0.675, p = 0.61	F(1,44) = 0.063, p = 0.80	F(1,44) = 0.000, p = 1.0
Within fan 1m while ON	F(4,42) = 0.729, p = 0.58	F(1,44) = 0.475, p = 0.49	F(1,44) = 0.116, p = 0.74
Within fan 1m once OFF	F(4,42) = 0.704, p = 0.59	F(1,44) = 1.159 p = 0.29	F(1,44) = 0.545, p = 0.46
Within door 1m while ON	F(4,42) = 0.994, p = 0.50	F(1,44) = 0.022, p = 0.88	F(1,44) = 1.011, p = 0.32
Within door 1m once OFF	F(4,42) = 0.676, p = 0.61	F(1,44) = 0.000, p = 1.0	F(1,44) = 0.017, p = 0.90
Laying down once OFF	F(4,42) = 0.302, p = 0.88	F(1,44) = 4.338, p = 0.043*	F(1,44) = 0.338, p = 0.56
Total time walking	F(4,42) = 1.280, p = 0.30	F(1,44) = 0.346, p = 0.56	F(1,44) = 2.241, p = 0.14
Total time eating	F(4,42) = 0.395, p = 0.81	F(1,44) = 0.686, p = 0.41	F(1,44) = 0.369, p = 0.55
Total time looking at door	F(4,42) = 0.231, p = 0.92	F(1,44) = 0.113, p = 0.74	F(1,44) = 1.501, p = 0.23
Total time sitting or lying down	F(4,42) = 0.679, p = 0.61	F(1,44) = 0.013, p = 0.91	F(1,44) = 0.599, p = 0.44
Total time looking at fan/board while ON and afterwards OFF	F(4,42) = 0.957, p = 0.44	F(1,44) = 0.009, p = 0.93	F(1,44) = 0.012, p = 0.91

* Seconds laying down was significant with sex, showing that male dogs spent more time in seconds laying down (M = 14.86, SD = 35.88) than female dogs (M = 0.77, SD = 2.70) after the fan was off.
